# Crystal Structure of Non-Structural Protein 10 from Severe Acute Respiratory Syndrome Coronavirus-2

**DOI:** 10.3390/ijms21197375

**Published:** 2020-10-06

**Authors:** Annika Rogstam, Maria Nyblom, Signe Christensen, Celeste Sele, Vladimir O. Talibov, Therese Lindvall, Anna Andersson Rasmussen, Ingemar André, Zoë Fisher, Wolfgang Knecht, Frank Kozielski

**Affiliations:** 1 Department of Biology & Lund Protein Production Platform, Lund University, Sölvegatan 35, 22362 Lund, Sweden; annika.rogstam@biol.lu.se (A.R.); maria.gourdon@biol.lu.se (M.N.); celeste.sele@biol.lu.se (C.S.); therese_k.lindvall@med.lu.se (T.L.); anna.rasmussen@biol.lu.se (A.A.R.); zoe.fisher@ess.eu (Z.F.); wolfgang.knecht@biol.lu.se (W.K.); 2 Department of Biochemistry and Structural Biology, Lund University, Naturvetarvägen 26, 22241 Lund, Sweden; signe.christensen@biochemistry.lu.se (S.C.); ingemar.andre@biochemistry.lu.se (I.A.); 3 BioMax, MAX IV Laboratory, Fotongatan 2, 22484 Lund, Sweden; vladimir.talibov@maxiv.lu.se; 4 Scientific Activities Division, Science Directorate, European Spallation Source ERIC, Box 176, SE-221 00 Lund, Sweden; 5 School of Pharmacy, University College London, 29-39 Brunswick Square, London WC1N 1AX, UK

**Keywords:** SARS CoV-2, COVID-19, non-structural proteins, nsp10, replication–transcription complex, RNA capping machinery

## Abstract

Severe Acute Respiratory Syndrome Coronavirus-2 (SARS-CoV-2), causing Coronavirus Disease 19 (COVID-19), emerged at the end of 2019 and quickly spread to cause a global pandemic with severe socio-economic consequences. The early sequencing of its RNA genome revealed its high similarity to SARS, likely to have originated from bats. The SARS-CoV-2 non-structural protein 10 (nsp10) displays high sequence similarity with its SARS homologue, which binds to and stimulates the 3′-to-5′ exoribonuclease and the 2′-O-methlytransferase activities of nsps 14 and 16, respectively. Here, we report the biophysical characterization and 1.6 Å resolution structure of the unbound form of nsp10 from SARS-CoV-2 and compare it to the structures of its SARS homologue and the complex-bound form with nsp16 from SARS-CoV-2. The crystal structure and solution behaviour of nsp10 will not only form the basis for understanding the role of SARS-CoV-2 nsp10 as a central player of the viral RNA capping apparatus, but will also serve as a basis for the development of inhibitors of nsp10, interfering with crucial functions of the replication–transcription complex and virus replication.

## 1. Introduction

A new coronavirus, Severe Acute Respiratory Syndrome Coronavirus-2, or SARS-CoV-2, emerged at the end of 2019 and is the causative agent of Coronavirus disease 2019, COVID-19. Since then, it has quickly spread, turning into a global pandemic that has, to date (4 October 2020), caused over 35 million infections and more than one million deaths (Johns Hopkins Coronavirus Resource Center: https://coronavirus.jhu.edu). There are now seven CoV strains that are known human pathogens. Four of these, HCoV-OC43, HCoV-229E, HCoV-NL63 and HCoV-HKU1, are self-limiting and only cause mild upper respiratory tract symptoms. Three of these viruses, SARS, Middle East Respiratory Syndrome (MERS) and SARS CoV-2, cause severe and life-threatening disease in humans. CoVs are generally thought to have a zoonotic origin and are believed to transmit from animals to humans. SARS and MERS had estimated mortality rates of ca. 9% and 33%, and they caused mostly localised outbreaks in China and Saudi Arabia, with low infection rates of about 8000 and 2220 infections, respectively [1,2]. By contrast, the new SARS-CoV-2 has rapidly spread across the globe, sparking efforts for limiting the spread of the virus by repurposing existing antiviral drugs and the development of vaccines. These efforts represent two strategies in finding treatment options to contain this novel virus strain [3].

CoVs are single-stranded RNA viruses with ca. 29 kb genomes. The genomic sequence for SARS-CoV-2 was published at the beginning of the year [4]. Functionally, very little is known about SARS-CoV-2, but significant detail can be inferred from work performed on the closely related variant, SARS. The Open Reading Frame 1ab (ORF1ab) represents two thirds of the entire genome and contains 15 non-structural proteins (nsps), termed nsp1 to nsp16 [5]. The remaining one third of the SARS-CoV-2 genome encodes the four structural proteins (Spike (S), Nucleocapsid (N), Envelope (E) and Membrane (M)), in addition to a variety of smaller accessory proteins, which may vary between the different members of the CoV family [6]. 

Many of the nsps are involved in viral replication and represent potential targets for antiviral drug discovery [7,8]. For example, SARS-CoV-2 nsp12, also known as RNA-dependent RNA polymerase (RdRp), forms a large multi-protein complex with nsp7 and nsp8, is involved in RNA replication and forms part of a much larger Replication–Transcription Complex (RTC) [9]. Remdesivir, formerly developed as a drug candidate to treat patients with Ebola, and ribavirin, which is used in the clinic to treat infections with Hepatitis C Virus (HCV), seem to be inhibitors of RdRp [10,11] and are currently being tested in multiple clinical trials against SARS-CoV-2 [12]. Remdesivir, a nucleoside analogue prodrug, has recently been shown to shorten hospitalisation for infected patients with a mild course of disease, but it does not seem to lower the mortality rate of patients with severe disease [13]. By contrast, dexamethasone has been shown to reduce mortality for the most severely affected patients [14]. There are no vaccines currently available against this viral pathogen. 

Another important protein involved in RNA replication is nsp10. Nsp10 is a small protein of ca. 140 amino acid residues that exists exclusively in viruses and not in prokaryotes or eukaryotes. In SARS, nsp10 was demonstrated to be essential for the stimulation of nsp14 and nsp16, acting primarily as a stimulatory and scaffolding protein. Nsp14 is a bimodular protein possessing unique N-terminal 3′-to-5′ exoribonuclease (ExoN) activity and C-terminal N7-methyltransferase (N7-MTase) activity. Nsp10 binds to and stimulates ExoN activity but does not seem to be required for the stimulation of the N7-MTase [15]. Nsp10 is also required for the stimulation of 2′-O-MTase activity in nsp16 [16], making nsp10 a central player in the essential RNA methylation machinery [17]. 

In a study of Murine Hepatitis Virus (MHV) nsp10, alanine-scanning mutagenesis and reverse genetics experiments were conducted. Out of eight mutations located at the nsp16–nsp10 interface, seven resulted in lethal and debilitated virus phenotypes, underscoring the essential function of nsp10 in CoV replication [18]. Another study involved peptide derivatives of nsp10 that were designed to interact with the nsp16 2′-O-MTase. These were demonstrated in a proof-of-principle study to inhibit the interaction between nsp10 and nsp16, interfering with the stimulation of nsp16 in vitro [19]. Subsequent optimisation led to the development of a peptide named TP29 that exerts inhibitory effects in MHV-infected mice in vivo [20]. This study unambiguously showed that targeting 2′-O-MTase activity through nsp10-derived peptides successfully suppressed SARS-CoV replication in vivo.

Due to the central role of nsp10 as an essential stimulator of 3′-to-5′ ExoN and N7-MTase activities in CoVs, we determined the crystal structure of this protein to a 1.6 Å resolution and investigated its biophysical behaviour to gain first insights into SARS-CoV-2 nsp10′s structure, oligomeric state and thermal stability. In this study, we also compare the crystal structure of SARS-CoV-2 nsp10 with nsp10s from other human pathogenic CoVs. Moreover, we relate its structure to its oligomeric state in solution. This work serves as a basis for discovering inhibitors that can disrupt the assembly of the RTC and, by extension, inhibit viral replication. 

## 2. Results and Discussion

### 2.1. SARS-CoV-2 nsp10 Is a Zinc-Finger Protein of the LIM Type Containing Two Zinc-Finger Motifs

**Figure 1:**
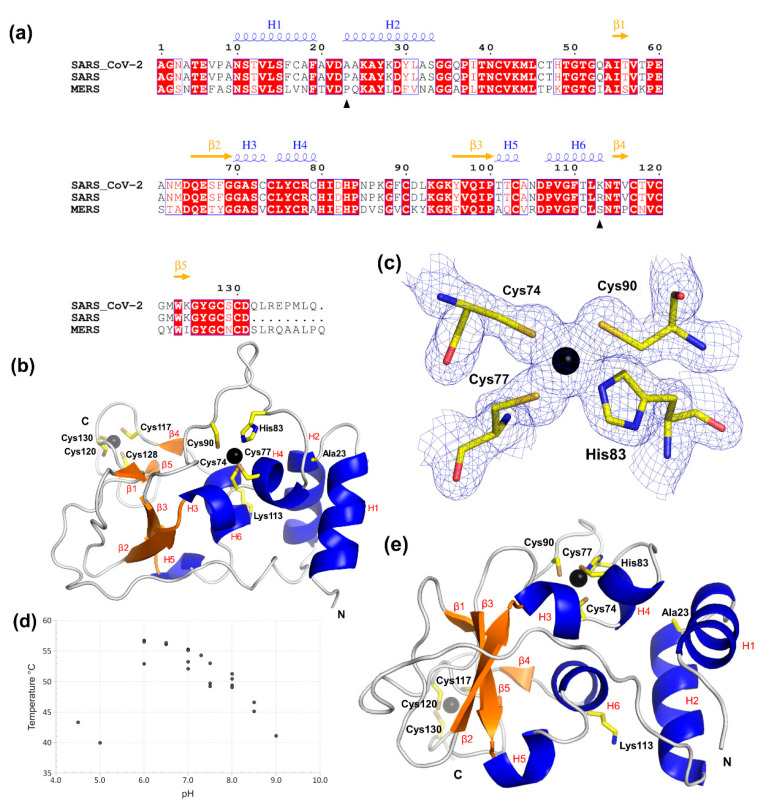
Overall structure of SARS-CoV-2 nsp10. (**a**) Alignment of nsp10 full-length protein sequences from MERS, SARS and SARS-CoV-2. Residues shaded in red are fully conserved, while residues with text in red indicate a change to a similar residue. Unshaded amino acids in black indicate non-conserved residues. The black arrows show the two residues that are different between SARS and SARS CoV-2. Figure was generated using ESPript, and the secondary structure elements were identified by STRIDE for nsp10 from SARS CoV-2 and are shown above the alignment [24,25]. (**b**) Illustration of nsp10′s overall structure with the two zinc fingers shown as a ball-and-stick model. Zn ions are shown as black spheres, and β-sheets and helices, assigned by STRIDE, are coloured orange and blue, respectively [25]. (**c**) The first zinc-finger motif in nsp10 from SARS-CoV-2. The 2F_o_-F_c_ electron density map is shown for this zinc site in blue mesh, contoured at 1.5 σ. Zinc-coordinating residues are labelled, and the zinc ion is shown as a black sphere. (**d**) Scatterplot of nanoDSF data. Nsp10 unfolding temperature T_m_ as a function of pH. (**e**) Residues that differ from SARS-CoV—Ala23 and Lys113—and cysteine and histidine side chains involved in the coordination of the zinc fingers are also shown as ball-and-stick models. N- and C-termini are indicated as single letters in black.

The structure of SARS-CoV-2 nsp10 was determined by molecular replacement using the SARS structure (Protein Data Bank (PDB) ID 2fyg) as a search model. Nsp10 crystallises in space group I2_1_3 with one molecule in the asymmetric unit. The refined model contains residues Asn10 to Asp131, and there are no flexible regions that could not be built. The data collection and refinement statistics are shown in Table 1. The sequence and secondary structure assignment are shown in Figure 1a.

The N-terminus of nsp10 is composed of two antiparallel α-helices (H1 and H2), which are connected via a long loop to the first β-strand, followed by β2 that forms a small β-sheet with β3 (Figure 1b). This β-sheet contains a large insertion folding into helices H3 and H4 containing the first zinc finger composed of three-cysteine and one-histidine side chains that coordinate the zinc ion (Figure 1c). Following β3, we observe a short helical turn, which has not been assigned in SARS nsp10 and which is located adjacent to α-helix H6. A long C-terminal loop region interrupted by the two short strands β4 and β5 houses the second zinc finger, composed of four cysteine side chains, which seems to be required to stabilise this region. The zinc-finger structure is C74-x_2_-C77-x_5_-H83-x_6_-C90 followed by C117-x_2_-C120-x_7_-C128-x-C130, indicating that nsp10 is a non-classical zinc-finger protein [21]. Zinc fingers are small protein structural motifs in which zinc ions are coordinated by a combination of cysteine and histidine side chains and are involved in many vital cellular processes. As no zinc was added during purification or to the crystallisation solution, the zinc ions present in nsp10 most likely came from expression in *E. coli*. To further confirm the presence of zinc ions, we carried out X-ray fluorescence (XRF) scanning experiments on one of the nsp10 crystals. The results from the XRF clearly reveal the zinc peak at 8.6 keV (K_α_ emission) (Figure S1). 

To further characterise nsp10, a stability scan over a range of different pH values was performed using nano differential scanning fluorimetry (nanoDSF). The resulting melting temperatures (T_m_) can roughly be divided into three groups (Figure 1d, Table S1). In the first group, below pH 6.0, the protein either yields an atypical melting curve or results in T_m_ values more than 10 degrees below the highest obtained T_m_ of 56.7 °C. In the second group, with pH values ranging from 6.0 to 8.0, the T_m_ values are 50 °C and above, whereas in the third group, with pH values above 8.0, nsp10 is, again, less stable. The different buffer systems kept at the same pH values show similar results, indicating that it is the pH itself and not the buffer type that is important for the stability of nsp10, in agreement with mechanisms of zinc coordination and folding at neutral pH [22]. To further confirm this effect, we conducted nanoDSF in the presence of two chelators, ethylenediaminetetraacetic acid (EDTA) and dipicolinic acid (DPA), at pH 8.0, at which the zinc fingers have been shown to be stable. Both chelators induced the loss of nsp10 stability (Table S2), likely due to the loss of zinc coordination, further confirming our previous results. 

### 2.2. SARS-CoV-2 nsp10 Exhibits Close Similarity to Its SARS Homologue

As SARS-CoV-2 is a new strain of SARS, this is reflected in the high sequence similarity of the non-structural proteins, in particular, nsp10. This high sequence similarity is also reflected in their structural similarity, with an RMSD. of 0.498 Å for all the atoms (PDB ID 2fyg) throughout the structures of the two nsp10 homologues. There are only two amino acid changes between the two SARS strains (Figure 1a,e). The first change, Pro23Ala, is located at the end of the loop connecting helices H1 and H2, in which the nonpolar, helix-initiator proline is substituted with a nonpolar alanine, thus maintaining the predominantly nonpolar environment. The second change, Arg113Lys, maintaining an amino acid with a positive charge, is located at the apex of α-helix H6. Whereas the Arg113 side chain in SARS displays a hydrogen-bond interaction with the side chain of Asp106, this interaction is no longer present with Lys113 in SARS-CoV-2. Although they are at distant locations in the protein sequence, they are close in space in the crystal structure (Figure 1e). Both represent very conservative changes; neither are in direct contact with their binding partners nsp14 and nsp16 and are therefore not expected to significantly influence nsp10 function.

### 2.3. Nsp10′s Absence in Prokaryotes and Eukaryotes Makes It a Unique Viral Protein

To identify proteins with a similar fold to SARS-CoV-2 nsp10, we used the DALI server [23] to extract structures deposited in the PDB that display a similar overall fold. Proteins with a significant *z*-score >20 included SARS nsp10 in the unbound form; SARS, MERS and SARS-CoV-2 nsp16–nsp10 complexes; and the SARS nsp14–nsp10 complex. There are no known structures with a fold similar to nsp10 in prokaryotes or eukaryotes, and similar proteins seem to be limited to CoVs. 

### 2.4. Characterisation of SARS-CoV-2 nsp10′s Behaviour in Solution Reveals Differences to Its SARS Homologue

Joseph et al. observed SARS nsp10 in the same space group as reported here, I2_1_3, reporting a monomer in the asymmetric unit but a dimer in solution, as was determined by size exclusion chromatography (PDB ID 2fyg) [26]. The high crystallographic symmetry made a number of dimer pairs possible, but it was not feasible, based on the structures alone, to identify which one could be relevant for the reported dimerization in solution. Another study crystallised a dodecamer of the SARS nsp10–nsp11 complex (nsp11 is a short peptide of 13 residues) in the asymmetric unit of the crystal [27]. The dodecamer has tetrahedral symmetry and is composed of a tetramer of trimers. 

**Figure 2:**
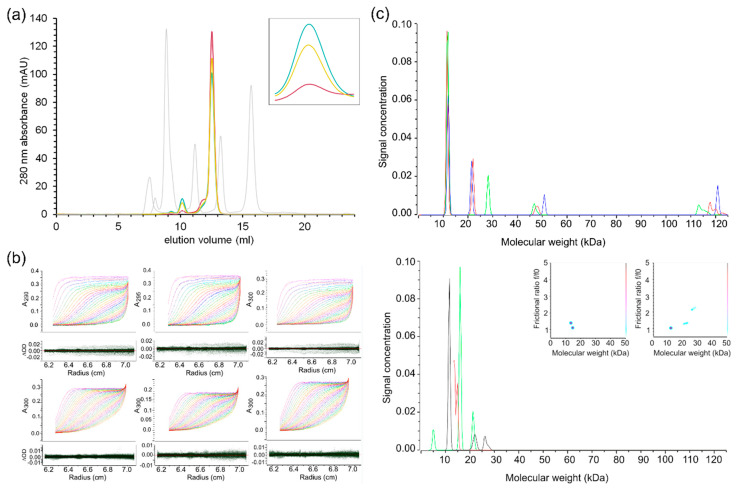
Biochemical characterization of SARS-CoV-2 nsp10. (**a**) Analytical size-exclusion chromatography data from a Superdex 75 Increase 10/300 GL column. Freshly purified nsp10 kept at 3 mg/mL (purple); freshly purified nsp10 concentrated to 55 mg/mL, stored for 20 h and then diluted to 3 mg/mL (yellow); freshly purified nsp10 concentrated to 55 mg/mL, stored for 44 h and then diluted to 3 mg/mL (cyan); calibration peaks, from left to right—blue dextran (column void volume, V_0_ = 7.51 mL), conalbumin (75 kDa, V_e_ = 8.85 mL), carbonic anhydrase (29 kDa, V_e_ = 11.16 mL), ribonuclease A (13.7 kDa, V_e_ = 13.22 mL), aprotinin (6.5 kDa, V_e_ = 15.68 mL)—are coloured in grey. The major nsp10 peak (V_e_ = 12.52 mL) corresponds to a molecular weight of 18.9 kDa (interpreted as a monomer), while the minor nsp10 peak (V_e_ = 10.14 mL) corresponds to a molecular weight of 44.2 kDa (interpreted as a dimer). Inset: dimer peak, close-up view. High protein concentrations and storage increase the dimer-to-monomer ratio. (**b**) Sedimentation velocity data for nsp10. Protein concentrations of 0.5, 1, 2 and 3 mg/mL were fitted simultaneously to shape and size distributions. Two-dimensional spectrum analysis refined by a genetic algorithm (red curves) fitted to sedimentation velocity data (rainbow curves) is shown for nsp10 at concentrations of 0.5 mg/mL from a 55 mg/mL stock (upper left panel, RMSD = 0.0030), 1 mg/mL from a 55 mg/mL stock (upper middle panel, RMSD = 0.0027), 2 mg/mL from a 55 mg/mL stock (upper right panel, RMSD = 0.0035), 1 mg/mL from a 3 mg/mL stock (lower left panel, RMSD = 0.0017), 2 mg/mL from a 3 mg/mL stock (lower middle panel, RMSD = 0.0015), and 3 mg/mL from a 3 mg/mL stock (lower right panel, RMSD = 0.0018). Residuals are shown in the lower bars. (**c**) Sedimentation velocity data were analysed individually by a two-dimensional spectrum analysis that simultaneously fits shape and size and was further refined with a genetic algorithm. The resulting molecular weight distributions are shown for data recorded for nsp10 prepared from a stock stored at 55 mg/mL (upper panel) and 3 mg/mL (lower panel) in red (0.5 mg/mL), green (1 mg/mL), blue (2 mg/mL) and black (3 mg/mL). Inserted in the lower panel is a pseudo-3D plot showing the frictional ratio, molecular weight and partial concentration for species present in 2 mg/mL (left) and 3 mg/mL (right) nsp10.

To determine the oligomeric state of SARS-CoV-2 nsp10 in solution, we subjected our nsp10 construct to analytical size-exclusion chromatography. The protein was taken directly from preparative size-exclusion chromatography and was concentrated to 3 mg/mL and analysed immediately, or concentrated to 55 mg/mL (the concentration used for crystallization) and incubated at 4 °C for 20 and 44 h before diluting it to 3 mg/mL. The samples were analysed on a Superdex 75 Increase 10/300 GL column, calibrated with proteins of known molecular weights. For all samples, one major and one minor peak was observed (Figure 2a). These peaks most likely correspond to monomeric and dimeric nsp10, although the molecular weights calculated using the calibration of the column deviated by approximately 30% from the expected values (Figure 2a). For the fresh protein kept at 3 mg/mL, the ratio between the dimer and monomer was 1:50, while for the protein kept at 55 mg/mL, the ratio increased to 1:15 after 20 h, and to 1:10 after 44 h, indicating that dimerization is concentration and storage dependent. To investigate the effects of long-term storage, concentrated nsp10 (55 mg/mL) stored for one month was diluted to 3 mg/mL and analysed on a Superdex 200 Increase 10/300 GL column. The results showed that after long-term storage at high concentration, aggregates form, and the amount of dimers increases substantially (Supplementary Figure S2). To elucidate if disulfide bond formation played a role in dimerization, the same run was repeated in the presence of a reducing agent (1 mM TCEP), but no difference in elution profile was observed (Supplementary Figure S2). 

Since the molecular weight of the nsp10 monomer calculated from the Superdex 200 Increase calibration deviated from the expected value (Supplementary Figures S2 and S3), we employed sedimentation velocity ultracentrifugation to further investigate the oligomeric state of nsp10 (Figure 2b,c). The protein was studied at concentrations of 0.5, 1, 2 and 3 mg/mL, and the shape and molecular weight distribution were estimated from the data (Figure 2b). Experiments were performed with samples prepared from nsp10 stored at 55 mg/mL and from freshly purified nsp10 at 3 mg/mL. The analysis suggested that the predominant species for both preparations at all concentrations is consistent with the theoretical molecular weight of monomeric nsp10 (13.3 kDa) (Figure 2b, Table S2). The second-most-populous species is consistent with dimeric nsp10 (26.6 kDa). However, freshly prepared nsp10 has a higher proportion of monomer (74–100%) compared to nsp10 stored at 55 mg/mL (53–68%). Species with higher oligomeric states were present at lower concentrations in nsp10 stored at 55 mg/mL, but there was no evidence of oligomeric states higher than dimers in freshly prepared nsp10. This is consistent with our size-exclusion chromatography (SEC) data, with monomeric nsp10 as the most prominent form in solution.

For 2 mg/mL of freshly prepared nsp10, we observed two species with molecular weights corresponding to monomeric nsp10. The two monomeric species both have molecular weights equivalent to the monomer observed at all other concentrations (Monomer 1). Whereas one of these monomeric species has hydrodynamic properties equivalent to Monomer 1, the frictional ratio and sedimentation coefficient shifted in the other monomeric species (Monomer 2) (Table S3). At 3 mg/mL nsp10, we observe two species with molecular weights corresponding to dimeric nsp10. One of the observed dimeric species has hydrodynamic properties corresponding to the dimer observed at all other concentrations except for 2 mg/mL of nsp10 prepared from Stock 2 (Dimer 1). The additional dimer (Dimer 2) is also observed at 2 mg/mL freshly prepared nsp10 and shows a downward shift in sedimentation coefficient and upward shift in frictional ratio compared to Dimer 1. The theoretical sedimentation coefficient for monomeric nsp10 was calculated using the program US-SOMO based on the crystal structure of nsp10 (Table S4). The theoretical sedimentation coefficient (1.9 S) and frictional ratio (1.2) are in excellent agreement with the measured values for Monomer 1 (1.9 S and 1.2, respectively). This indicates that in solution, Monomer 1 adopts the conformation described by the crystal structure. The observed upward shift in the frictional ratio and downward shift in the sedimentation coefficient for Monomer 2 suggest that the shape differs from Monomer 1. This can be due to, for example, a conformational change, differences in the degree of solvation or the presence of ions, potentially related to the binding and release of zinc ions to and from nsp10. In conclusion, although structurally very similar, SARS-CoV-2 and SARS differ by the absence [5] and presence of nsp11, which may explain the higher oligomeric association for the SARS nsp10–nsp11 complex. Whereas SARS-CoV-2 nsp10 is predominantly a monomer in solution, the determination of the oligomeric state of SARS nsp10 will require additional experiments in order to compare both proteins.

### 2.5. Comparison between the Unbound and Complex-Bound Forms of nsp10 Reveals Subtle Differences

**Figure 3:**
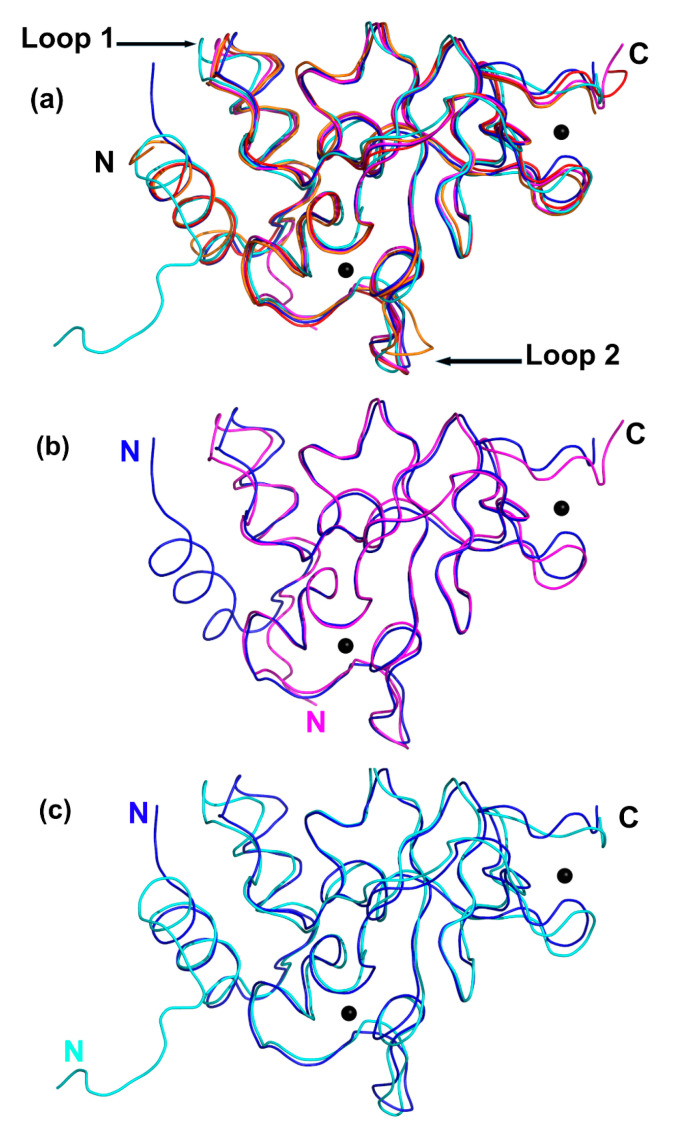
Structural overlays of unbound nsp10 (this work) compared to bound nsp10 from SARS, MERS and SARS-CoV-2. (**a**) Overlay of SARS-CoV-2 nsp10 (blue), SARS-CoV-2 nsp10–nsp16 complex (magenta, PDB ID 6w75), unbound SARS nsp10 (red, PDB ID 2fyg), SARS nsp10–nsp14 complex (cyan, PDB ID 5c8u) and MERS nsp10–nsp16 (orange, PDB ID 5yn5). Zn ions are shown as grey spheres. Variable Loops 1 and 2 are indicated by black arrows. (**b**) Overlay of nsp10 (blue) and nsp10 from nsp10–nsp16 complex from SARS-CoV-2 (magenta; PDB ID 6w75). (**c**) Overlay of nsp10 (blue) and nsp10 from nsp10–nsp14 complex from SARS (cyan; PDB ID 5c8u).

Very recently, coordinates of the SARS-CoV-2 nsp16–nsp10 complex with and without S-adenosyl-_L_-methionine (SAM) have been deposited in the PDB (PDB IDs 6w4h, 6w75 and 6w61) [27,28]. SAM acts as a co-factor, providing the methyl group for the methylation of the first nucleotide of the viral RNA catalysed by the 2′O-MTase. This cap methylation protects the viral RNA from recognition by the host immune system and subsequent destruction [29]. We will therefore compare our unbound SARS-CoV-2 nsp10 with the bound nsp10 determined in the SARS nsp10–nsp14 (PDB ID 5c8u) [30] and nsp10–nsp16 complexes from SARS-CoV-2 and MERS (PDB ID 5yn5). A series of overlays indicate strong similarities with very subtle differences between the unbound nsp10 (this work) and nsp10 in complex with nsp14 (SARS) and nsp16 (MERS and SARS-CoV-2) (Figure 3a). All the nsp10 structures are structurally highly similar and can be overlaid with an average RMSD less than 1.0 Å over all atoms. There are two areas that correspond to surface loops that slightly vary (displacement between 2 and 3 Å) between the different structures (Figure 3a). Loop 1, composed of residues 32–35, following α-helix H2, is close to where nsp14 would bind, while Loop 2, composed of residues 85–88, located between helix H4 and β3, is not in close proximity to nsp14 or nsp16 [31]. This variation could be due to differences in crystal packing or crystallisation conditions. 

Figure 3b shows the overlay of unbound nsp10 and nsp16-bound nsp10 from SARS-CoV-2. One significant difference is the change in the N-terminus, which includes helix H1 in unbound nsp10 but is absent in the nsp16–nsp10 complex, probably due to how the expression plasmid was designed. Unbound nsp10 from SARS-CoV-2 includes an additional 11 residues, and these adopt an α-helix (H1) and are seen in the opposite direction from the N-terminus of nsp10 bound to nsp16. Helix H1 is close to the N-terminus and is also seen in the MERS nsp10–nsp16 complex and in both the nsp14-bound and unbound forms seen in SARS (Figure 3a,c). The N-terminal helix H1 is in close contact with nsp14, and the additional residues prior to the helix in the SARS nsp10–nsp14 structure can be seen to interact with nsp14. In unbound nsp10, these residues are not in contact with any molecules, yet they adopt the same overall structure. The residues in unbound nsp10 that comprise the interface with either nsp14 or nsp16 reveal no major rearrangements compared to their side chain conformations in complex with nsp14 or nsp16. In the unbound form of nsp10, there are solvent molecules that act as placeholders for the amino acid side chain interactions established with residues from either nsp14 or nsp16. In conclusion, unbound nsp10 does not display major conformational changes compared to its conformation when bound to either nsp14 or nsp16, with the exception of a slight rearrangement of Loop 1 to accommodate nsp14 (black arrow in Figure 3a).

### 2.6. SARS-CoV-2 nsp10, as a Central Player, Represents a Potential Antiviral Drug Target

In SARS-CoV, nsp10 has been shown to be a central player in RNA replication by interacting with and stimulating the activities of the ExoN domain of nsp14 and the 2′-O-MTase activity of nsp16 [32] shown to be essential for CoV replication. Interestingly, the ExoN activity has also been shown to possess RNA proofreading capabilities and may be responsible for the lower mutation rates observed in CoV compared to other RNA viruses [33]. This proofreading capability has also been proposed to be responsible for the excision of nucleoside-based inhibitors from replicating RNA catalysed by RdRp that would otherwise lead to the suppression of RNA replication and virus survival [34]. Some of these nucleoside-based inhibitors are currently in a multitude of clinical trials to investigate their inhibitory potential against SARS-CoV-2 [35]. Many of the non-structural proteins involved in viral replication are also potential drug targets, but this area is under-researched [7,8]. 

We determined the crystal structure and behaviour in solution of SARS-CoV-2 nsp10 in its unbound form. The results, in particular, the high resolution of the structure at a 1.6 Å resolution, will serve as a starting point for inhibitor/fragment screening and structure-guided drug design. As nsp10 seems to be essential for viral replication in coronaviruses and has, to date, only been identified in viruses but not in prokaryotes or eukaryotes, it represents a potential antiviral drug target. Screening using the unbound nsp10 form could lead to the identification of allosteric as well as protein–protein interaction inhibitor-binding pockets. The goal is to interfere with the binding of nsp10 to nsp14 and nsp16, thus supressing the stimulation of the essential 3′-to-5′ ExoN and 2′-O’-MTase functions.

## 3. Materials and Methods

### 3.1. Design of the SARS-CoV-2 nsp10 Expression Plasmid 

The protein sequence of SARS nsp10 (NP_828868, 139 residues) was aligned with the Open Reading Frame 1ab (ORF1aB) of SARS-CoV-2 (GenBank: MN908947.3) to identify the location and sequence for nsp10. SARS nsp10, located between Ala4231 to Gln4369 of pp1a/pp1ab, corresponds to Ala4254 to Gln4392 in SARS-CoV-2. These two aligned sequences were subsequently compared to the SARS nsp10 structure (PDB ID 2fyg) as well as the nsp14–nsp10 and nsp16–nsp10 complexes, respectively, to further delineate the coding sequence for SARS-CoV-2. This sequence comparison was subsequently used as a basis to synthesise (Genscript, Leiden, Netherlands) a DNA sequence codon optimised for recombinant expression in *Escherichia coli*. We also added in-frame *NcoI* restriction sides at the 5′ and a stop codon followed by an *XhoI* restriction site at the 3′ end of the DNA sequence. The DNA sequence was double restricted with *NcoI* and *XhoI* and subcloned into a modified ppSUMO expression vector, in which *NcoI* and *XhoI* restriction sites had previously been introduced directly following an Ulp1 cleavage site. This nsp10 expression plasmid was named ppSUMO-nsp10, corresponding to residues Asn4264 (renumbered to residue Asn10 for convenience) to Gln4385 (123 residues) of SARS-CoV-2, respectively. Due to the cloning strategy, the three unspecific residues Thr7, Met8 and Gly9 are located at the N-terminus of the protein.

### 3.2. Nsp10 Expression and Purification

*E. coli* TUNER (DE3) cells (Novagen, Darmstadt, Germany) carrying the ppSUMO-nsp10 expression plasmid were grown in 9 L of BD Difco LB Broth Miller supplemented with 50 µg/mL kanamycin in 2.5 L Full-Baffle Tunair Shake Flasks (IBI Scientific, Dubuque, Iowa, USA), with 1 L/flask, at 18 °C and 250 rpm. At OD_600_ = 0.7, the expression of the nsp10 gene was induced by the addition of 1 mM isopropyl-d-thiogalactopyranoside (IPTG), and growth was continued for 20 h before the cells were harvested at 8000× *g* for 20 min at 4 °C. Cell pellets were resuspended in 50 mM NaPO_4_, 300 mM NaCl and 20 mM imidazole at pH 8.0, supplemented with Complete Protease Inhibitor (EDTA free) (Roche, Stockholm, Sweden). The cells were lysed by passing the cell suspensions twice through a French pressure cell at 18,000 psi. Cell debris was removed by ultracentrifugation at 100,000× *g* for 60 min at 4 °C. The clarified lysate was loaded onto a 5 mL HisTrap HP column (GE Healthcare, Uppsala, Sweden) connected to an ÄKTA Pure chromatography system (GE Healthcare, Uppsala, Sweden). The column was washed with 20 column volumes of 50 mM NaPO_4_, 300 mM NaCl and 20 mM imidazole at pH 8.0, and the bound protein was eluted with a 0–100% gradient of 50 mM NaPO_4_, 300 mM NaCl and 500 mM imidazole at pH 8.0 over 20 column volumes. Peak fractions were pooled and digested with SenP2 protease in the presence of 1 mM dithriotheitol (DTT), while dialyzing against 50 mM NaPO_4_, 300 mM NaCl, 20 mM imidazole and 1 mM DTT at pH 8.0. The digested protein was loaded onto a 5 mL HisTrap HP column connected to an ÄKTA Pure chromatography system, and the flow through was collected, concentrated and loaded onto a HiLoad 26/600 Superdex 75 pg column (GE Healthcare, Uppsala, Sweden) connected to an ÄKTA Purifier chromatography system (GE Healthcare, Uppsala, Sweden) with 50 mM Tris and 150 mM NaCl at pH 8.0 as the running buffer. Peak fractions containing pure nsp10 were pooled for subsequent experiments (Figure S3). All purification steps were performed at 4 °C. The protein was concentrated using Amicon Ultra centrifugal filter units (Millipore, Darmstadt, Germany). The protein concentration was measured in a Nanodrop spectrophotometer using the theoretical extinction coefficient of nsp10 calculated using the Expasy ProtParam tool. The protein was analysed on Any kD Criterion TGX gels (BioRad, Solna, Sweden) stained with BioSafe Coomassie (BioRad, Solna, Sweden). 

### 3.3. Crystallisation of nsp10

Nsp10 was concentrated to 55 mg/mL prior to crystallisation. Screening against commercial screens was performed with a Mosquito (TTP Labtech, Melbourn, UK) crystallisation robot for droplets with a final volume of 300 nL against a reservoir volume of 40 μL. SwissSci plates were used, and three different drops were set up with different protein/precipitant ratios (100:200, 100:100 and 200:100 nL). After several days of incubation at 20 °C, crystals appeared in the JCSG+ condition G12, containing 3 M sodium chloride and 0.1 M Bis-Tris buffer at pH 5.5, with a volumetric ratio of 200:100 of protein/precipitant for nsp10. The cubic crystals grew to a size of ~10 µm. Higher-resolution crystals were obtained by using a second batch of protein concentrated at 58 mg/mL and an optimization screen. The best crystals grew to a size of ~125 µm in about 6 days under 20 °C incubation in 2.2 M sodium chloride and 0.1 M Bis-Tris buffer at pH 6.5, with a ratio of 200:100 nL of protein/precipitant. The crystals were harvested and briefly cryoprotected with reservoir solution supplemented with 20% glycerol before being cryocooled in liquid nitrogen. 

### 3.4. Data Collection, Structure Determination and Refinement

Diffraction data for nsp10 were collected at the BioMAX beamline at the MAX IV laboratory (Lund, Sweden). For measurements, the beam size was adjusted to 50 microns to cover the entire crystal. Data were collected by fine-slicing with an oscillation range of 0.1°, 360° total. The data were processed with XDS [36] and Aimless to a resolution of 1.58 Å. The structure was solved by molecular replacement with SARS nsp10 as a search model (PDB ID 2fyg) using PHASER of the PHENIX suite [37]. Electron density and difference density maps, all σA-weighted, were inspected, and the model was improved using *Coot* [38]. The structure was refined with PHENIX [39]. The calculation of R_free_ used 5% of the data. Crystallographic and refinement statistics are provided in Table 1. In the nsp10 structure, there are no flexible regions, and the structure covers residues Asn10 to Asp131 of nsp10 and contains two zinc fingers. 

As no zinc was added during the purification or to the crystallisation buffer, an X-ray fluorescence scan was performed on nsp10 crystals. The measurements were taken at 12 keV when the synchrotron was operating at 50 mA. The beam aperture was set to 50 microns, and 1 s exposures were recorded. Figure S1 in the supplementary material shows the raw fluorescence spectra collected, and the Kα emission peak of Zn was clearly visible at ~8.6 keV. 

### 3.5. Analytical Size Exclusion Chromatography

A Superdex 75 Increase 10/300 GL column or a Superdex 200 Increase 10/300 GL column (GE Healthcare, Uppsala, Sweden) connected to an ÄKTA Pure chromatography system (GE Healthcare, Uppsala, Sweden) was equilibrated with 2 column volumes (CV) of 50 mM Tris, pH 8.0, and 150 mM NaCl. Then, 100 µL of nsp10 was injected, and elution was performed at a flow of 0.5 mL/min for 1.2 CV at 4 °C. One additional run was carried out in the presence of 1 mM TCEP (tris(2-carboxyethyl)phosphine). Calibration was carried out in 50 mM Tris, pH 8.0, and 150 mM NaCl, using proteins from the GE Healthcare gel filtration calibration kits. Conalbumin, carbonic anhydrase, ribonuclease A and aprotinin were used for the Superdex 75 column. Ferritin, aldolase, conalbumin, ovalbumin, carbonic anhydrase, ribonuclease A and aprotinin were employed for the Superdex 200 column. Blue dextran 2000 (GE Healthcare, Uppsala, Sweden) was used to determine the void volume of both columns. 

### 3.6. Thermal Shift Assays

The same preparation of nsp10 as was set up in the crystallisation experiments was used for the nanoDSF experiments, screening a pH range from 4.5 to 9.0. In order to reduce the effect of the original buffer, the sample was diluted 18-fold to a stock of 3 mg/mL in a solution containing only 150 mM NaCl. In a final volume of 15 µL, 1 µL of 3 mg/mL protein stock was mixed with 3 µL of various pH buffer stock solutions (500 mM; Rubic additive screen, MD1-97, Molecular Dimensions, Sheffield, UK) and 11 µL of 191 mM NaCl solution, yielding final concentrations of 0.2 mg/mL of protein, 100 mM buffer and 150 mM NaCl. We also investigated the effects of two chelators, EDTA and DPA, on the stability of zinc-ion-containing nsp10. In a final volume of 15 µL, 2 µL of 2.2 mg/mL protein stock was mixed with 13 µL of 50 mM Tris HCl, pH 8.0, and 150 mM NaCl with added EDTA or DPA at various concentrations, yielding final concentrations of 0.3 mg/mL of protein in the presence of 1, 10 or 100 mM concentrations of the chosen chelating agent. Control measurements without any chelating agent were also conducted. The samples were loaded into a Prometheus NT.48 (Nanotemper, München, Germany) and measured in triplicate using standard-grade capillaries. The samples were heated at 1 °C/min, from 20 to 95 °C, and the unfolding of the protein was analysed according to the ratio of the wavelengths measured at 350 and 330 nm (tryptophan/tyrosine shifts) and with a laser power of 20%. From the resulting curves, the thermal unfolding transition midpoint T_m_ (°C), at which half of the protein population is unfolded, could be extracted, and the mean of the triplicate values for each pH was calculated (Table S1).

### 3.7. Analytical Ultracentrifugation

Analytical ultracentrifugation was performed with a Beckmann Optima 1 analytical ultracentrifuge with absorption optics using a An60Ti rotor. Nsp10 in 50 mM Tris, pH 8.0, and 150 mM NaCl at concentrations of 0.5, 1, 2, 3 and 5 mg/mL was loaded into two-sector cells made of charcoal-Epon. Sedimentation velocity experiments were run at a rotor speed of 55,000 or 45,000 rpm at 15 °C. Then, 190 radial scans were recorded in 20 s intervals at 280, 290, 295 and 300 nm. The sedimentation velocity data were analysed using the UltraScan III software [40]. Data quality issues prevented the analysis of the data set recorded for the 0.5 and 5 mg/mL nsp10 concentrations prepared from a fresh 3 mg/mL stock and a stock stored at 55 mg/mL, respectively. Before fitting a model to the velocity data, radial and time-invariant noise was subtracted. The data sets were analysed individually by a two-dimensional spectrum analysis that simultaneously fitted the shape and molecular weight distribution and was refined using a genetic algorithm [41,42]. Monte Carlo error analysis was carried out to obtain confidence intervals with 50 iterations [43]. For the simulation of hydrodynamic properties, the theoretic sedimentation coefficient (s_20,w_) and frictional ratio (f/f_0_) for the crystal structure of nsp10 were computed using the software US-SOMO [44]. The simulation was carried out using the SoMo bead model without overlap removal coupled with Zeno computations using default settings.

## 4. Conclusions

SARS-CoV-2 has triggered a global pandemic, leading to devastating consequences for health-care and social–economic systems. The control of this pandemic and a return to normal life will necessitate the development of efficient vaccines and antiviral drugs, requiring progress in the understanding of SARS-CoV-2 biology. Coronaviruses possess structural and accessory proteins as well as non-structural proteins (nsps), many of which are implicated in the replication of the viral genome and therefore essential for viral viability. Nsp10, as a central player, stimulates the 3′-to-5′ exoribonuclease and 2′-O-methyltransferase activities of nsp14 and nsp16, making it a crucial player in both RNA proofreading and RNA capping. Nsp10 is, therefore, considered as a drug target for coronaviruses with the potential to stop viral replication. Providing structural information on nsp10 is therefore an important first step to enable structure-guided drug discovery for this target.

## Figures and Tables

**Table 1:** Data collection, structure determination and refinement statistics for nsp10 from SARS-CoV-2. Data in parenthesis correspond to the highest resolution shell.

Data Collection and Refinement Statistics	SARS-CoV-2 nsp10 (PDB ID 6ZPE)
Wavelength (Å)	0.976
Resolution range (Å)	75.39–1.58 (1.61–0.58)
Space group	I2_1_3
Unit cell parameters (Å; °)	*a* = *b* = *c* = 106.62 Å; α = β = γ = 90
Matthew coefficient (Å^3^/Da)	3.81
Molecules per asymmetric unit	1
Total reflections	277,522 (14,080)
Unique reflections	27,706 (1391)
Multiplicity	10.0 (10.1)
Completeness (%)	99.9 (100.0)
Mean I/sigma(I)	21.1 (2.0)
Wilson B-factor	29.95
R-meas (%)	4.7 (128.3)
CC_1/2_ (%)	99.9 (70.9)
Reflections used in refinement	27,702 (2782)
R_cryst_/R_free_ (%)	15.2/16.0
Total no. of non-hydrogen atoms (protein)	1122
No. of protein/ligand/solvent residues	127/11/169
RMSD bond lengths, bond angles (Å; °)	0.012/1.09
Ramachandran favored/allowed/outliers/rotamer outliers (%)	98.4/1.6/0.0/0.0
Clashscore	2.7
Average B-factor/protein/ligands/solvent	38.0/35.9/55.8/48.2

## Supplementary Materials

Supplementary materials can be found at https://www.mdpi.com/1422-0067/21/19/7375/s1. Table S1. Nsp10 unfolding temperatures determined by DSF as a function of the pH. Table S2. Nsp10 unfolding temperatures determined by DSF in the presence of chelating agents EDTA and DPA. Table S3. Molecular weight distribution estimates from a genetic algorithm refined 2 dimensional spectrum analysis fitting frictional ratio and sedimentation coefficient from sedimentation velocity data. Table S4. Sedimentation coefficient in water at 20 °C (S_20,w_) and frictional ratio for monomeric and dimeric species observed in sedimentation velocity experiments. Figure S1 X-Ray Fluorescence scan of the Nsp 10 crystal from which the data was acquired. Figure S2. Analytical size exclusion chromatography data from a Superdex 200 Increase 10/300 GL column. Figure S3. Chromatogram from the last step in the purification of nsp10: size exclusion chromatography on a HiLoad 26/600 Superdex 75 pg column (GE Healthcare).

## Abbreviations

Å	Angstrom
CoV	Coronavirus
DPA	Dipicolinic acid
DTT	Dithiothreitol
EDTA	Ethylenediaminetetraacetic acid
ExoN	3′-to-5′ exoribonuclease
IPTG	Isopropyl-d-thiogalactopyranoside
MERS	Middle East Respiratory Syndrome
MHV	Murine Hepatitis virus
MTase	Methyltransferase
Nsp	Non-structural protein
ORF	Open reading frame
PDB	Protein Data Bank
RdRp	RNA-dependent RNA polymerase
RMSD	Root Mean Square Deviation
RTC	Replication transcription complex
SAM	S-adenosyl-L-methionine
SARS	Severe Acute Respiratory Syndrome
SEC	Size-Exclusion Chromatography
TCEP	Tris (2-carboxyethyl) phosphine
