# Supplementary material for: Crystal Structure of Non-Structural Protein 10 from Severe Acute Respiratory Syndrome Coronavirus-2
Source: Int J Mol Sci. 2020 Oct 6;21(19):7375. doi: 10.3390/ijms21197375 (PMC7583907; doi:10.3390/ijms21197375)

## Supplemental Information

**Table S1.** Nsp10 unfolding temperatures determined by DSF as a function of the pH. The mean $T_m$ values are calculated from measurements conducted in triplicate and the error is indicated.

| pH | Mean $T_m$ ± SE [°C] | Buffer component |
|---|---|---|
| 4.5 | 43.3 ± 0.22 | Sodium acetate |
| 5.0 | 40.0 ± 0.21 | Citrate |
| 6.0 | 56.7 ± 0.06 | MES |
| 6.0 | 56.5 ± 0.02 | Potassium phosphate |
| 6.0 | 52.9 ± 0.06 | Citrate |
| 6.5 | 56.1 ± 0.01 | Bis-Tris |
| 6.5 | 56.3 ± 0.02 | MES |
| 7.0 | 52.1 ± 0.03 | Sodium phosphate |
| 7.0 | 53.3 ± 0.01 | Potassium phosphate |
| 7.0 | 55.3 ± 0.01 | HEPES |
| 7.0 | 55.1 ± 0.01 | MOPS |
| 7.3 | 54.3 ± 0.05 | Ammonium acetate |
| 7.5 | 53.0 ± 0.02 | Tris-HCl |
| 7.5 | 49.7 ± 0.01 | Sodium phosphate |
| 7.5 | 49.2 ± 0.11 | Imidazole |
| 8.0 | 49.3 ± 0.01 | HEPES |
| 8.0 | 49.0 ± 0.01 | Tris-HCl |
| 8.0 | 51.3 ± 0.00 | Tricine |
| 8.0 | 50.4 ± 0.01 | BICINE |
| 8.5 | 46.6 ± 0.03 | BICINE |
| 8.5 | 45.1 ± 0.03 | Tris-HCl |
| 9.0 | 41.1 ± 0.06 | CHES |

**Table S2.** Nsp10 unfolding temperatures determined by DSF in the presence of chelating agents EDTA and DPA. The mean $T_m$ value is calculated from measurements carried out in triplicate and the error is indicated.

| **Mean $T_m$ ± SE [°C]** | **Added buffer component** |
|---|---|
| 49.3 ± 0.01 | None |
| 46.1 ± 0.00 | 1 mM EDTA |
| 44.8 ± 0.01 | 10 mM EDTA |
| 42.9 ± 0.01 | 100 mM EDTA |
| 48.7 ± 0.03 | 1 mM DPA |
| 44.0 ± 0.01 | 10 mM DPA |
| 37.2 ± 0.02 | 100 mM DPA |

**Table S3.** Molecular weight distribution estimates from a genetic algorithm refined 2 dimensional spectrum analysis fitting frictional ratio and sedimentation coefficient from sedimentation velocity data. The Mean and standard deviation for the monomer and dimer are calculated based on the estimated molecular weight for six models. n.d., not determined.

| | **Monomer [%]** | | **Dimer [%]** | | **Higher oligomer [%]** | | **RMSD** | |
|---|---|---|---|---|---|---|---|---|
| Conc. (mg/mL) | Stock 1 | Stock 2 | Stock 1 | Stock 2 | Stock 1 | Stock 2 | Stock 1 | Stock 2 |
| 0.5 | 62 | n.d. | 21 | n.d. | 17 | n.d. | 0.0030 | n.d. |
| 1.0 | 68 | 74 | 16 | 26 | 14 | 0 | 0.0027 | 0.0017 |
| 2.0 | 53 | 100 | 23 | 0 | 24 | 0 | 0.0035 | 0.0015 |
| 3.0 | n.d. | 76 | n.d. | 24 | n.d. | 0 | n.d. | 0.0018 |
| $M_w$ (kDa) | $12.6 \pm 1.7$ | | $23.4 \pm 2.6$ | | 47-120 | | | |

**Table S4.** Sedimentation coefficient in water at 20°C ($S_{20,w}$) and frictional ratio for monomeric and dimeric species observed in sedimentation velocity experiments. The theoretic hydrodynamic properties are calculated based on the crystal structure of nsp10 using the software US-SOMO.

| | **$S_{20,w}$ (S)** | **$f/f_0$** |
|---|---|---|
| Monomer theoretic | 1.9 | 1.2 |
| Monomer 1 | 1.9 ± 0.1 | 1.1 ± 0.1 |
| Monomer 2 | 1.5 | 1.3 |
| Dimer 1 | 2.7 ± 0.2 | 1.2 ± 0.1 |
| Dimer 2 | 1.45 ± 0.1 | 2.2 ± 0.1 |

**Figure S1 X-Ray Fluorescence scan of the Nsp 10 crystal from which the data was acquired.** Several major peaks are visible: chloride (2.6 KeV), iron (6.4 KeV) and zinc (8.6 KeV $\alpha_1$ and 9.6 KeV $\beta_1$) and scattered photons from the measuring energy (around 12 KeV). Another scan was run on the mother liquor of that crystal and was displaying the same peaks, except for the zinc ones, which are a unique feature of the crystal.

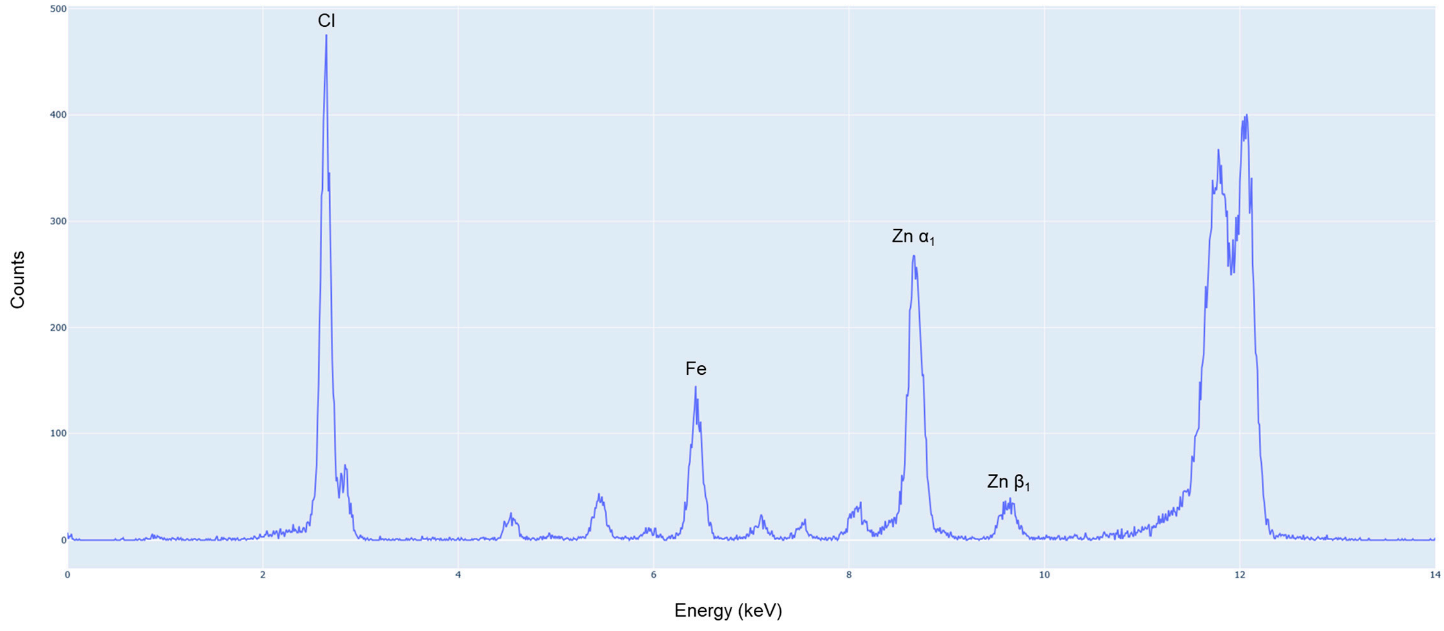

**Figure S2 Analytical size exclusion chromatography data from a Superdex 200 Increase 10/300 GL column.** Yellow: nsp10 stored at 55 mg/ml for one month, diluted to 3 mg/ml. Green: nsp10 stored at 55 mg/ml for one month, diluted to 3 mg/ml, run in the presence of 1 mM TCEP; grey: calibration runs, peaks from left to right: blue dextran (column void volume, $V_0$ = 8.30 ml), ferritin (440 kDa, $V_e$ = 10.44), aldolase (158 kDa, $V_e$ = 12.77 ml), conalbumin (75 kDa, $V_e$ = 14.21 ml), ovalbumin (44 kDa, $V_e$ = 15.14 ml), carbonic anhydrase (29 kDa, $V_e$ = 16.28 ml), ribonuclease A (13.7 kDa, $V_e$ = 17.66 ml), aprotinin (6.5 kDa, $V_e$ = 19.43 ml). The elution volume of the major nsp10 peak ($V_e$ =16.74 ml) corresponds to a molecular weight of 22.4 kDa, while the middle peak ($V_e$ = 15.40 ml) corresponds to 42.3 kDa, and the earliest peak elutes in the column void volume. The addition of a reducing agent does not influence the amount of dimers and aggregates.

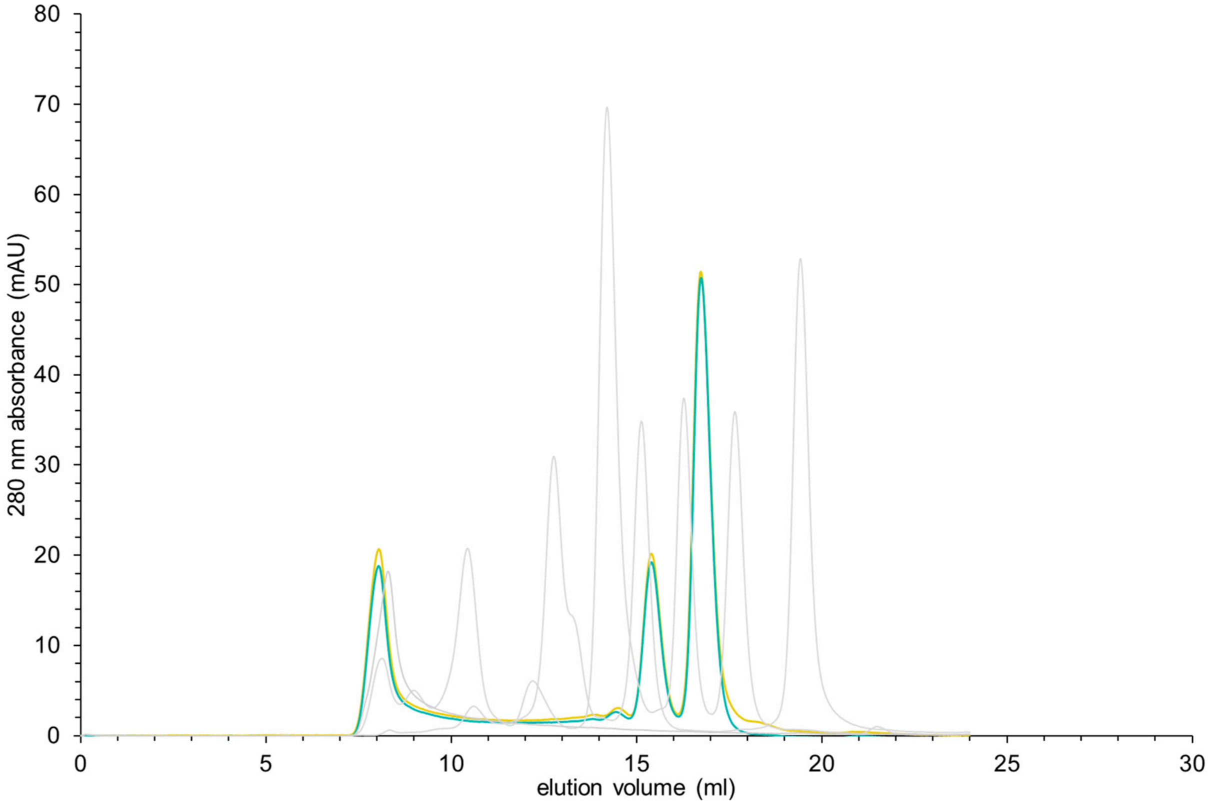

**Figure S3**. Chromatogram from the last step in the purification of nsp10: size exclusion chromatography on a HiLoad 26/600 Superdex 75 pg column (GE Healthcare). The major peak fractions were pooled (double arrow) and 2 µl of each fraction was analyzed on SDS-PAGE.

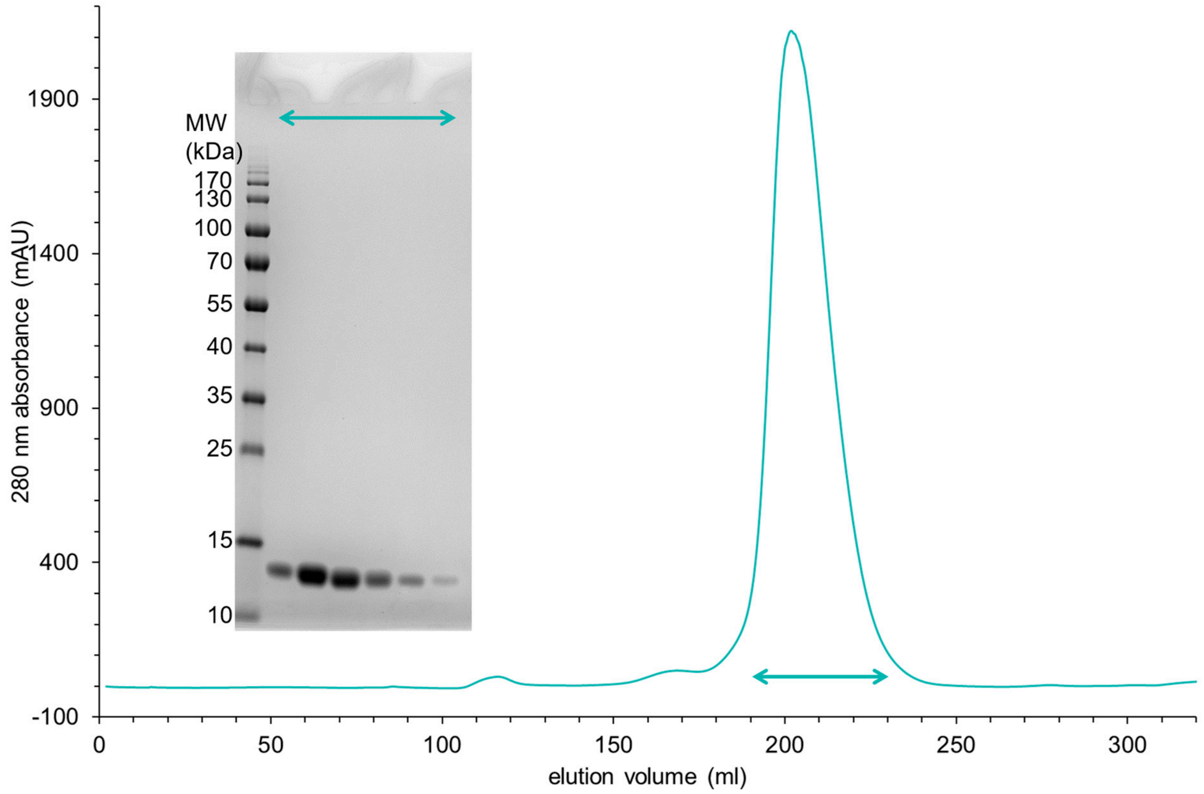

Supplement: Supplementary file 1 [file ijms-21-07375-s001.pdf]
